# Graphdiyne‐Induced Iron Vacancy for Efficient Nitrogen Conversion

**DOI:** 10.1002/advs.202102721

**Published:** 2021-11-07

**Authors:** Yan Fang, Yurui Xue, Lan Hui, Huidi Yu, Chao Zhang, Bolong Huang, Yuliang Li

**Affiliations:** ^1^ Institute of Chemistry Chinese Academy of Sciences Beijing 100190 P. R. China; ^2^ University of Chinese Academy of Sciences Beijing 100049 P. R. China; ^3^ Science Center for Material Creation and Energy Conversion Institute of Frontier and Interdisciplinary Science School of Chemistry and Chemical Engineering Shandong University Jinan 250100 P. R. China; ^4^ Department of Applied Biology and Chemical Technology the Hong Kong Polytechnic University Hung Hom Kowloon Hong Kong SAR 999077 P. R. China

**Keywords:** electrocatalytic nitrogen fixation to ammonia, graphdiyne, iron vacancy generation, structure optimization

## Abstract

An iron vacancy‐rich ferroferric oxide/graphdiyne heterostructure (IVR‐FO/GDY) is rationally designed and fabricated for high‐efficiency electrocatalytic nitrogen fixation to ammonia (ENFA). Experimental and theoretical results show that the GDY‐induced iron vacancies in IVR‐FO/GDY promote the electrocatalysis, and activate the local O sites to transfer electrons towards GDY to boost ENFA, resulting in promising electrocatalytic performances with a highest ammonia yield (Y_NH3_) of 134.02 µg h^−1^ mg_cat._^−1^ and Faradaic efficiency (FE) of up to 60.88%, as well as the high long‐term stability in neutral electrolytes. The cationic vacancy activation strategy proposed in this work has strong general and universal guiding significance to the design of new efficient electrocatalysts for various electrochemical energy conversion reactions. Such defect engineering may be used efficiently in electrocatalysis, leading to the development and progress of energy industry.

## Graphical abstract

A new strategy is proposed for the rational design and synthesis of iron vacancy‐rich ferroferric oxide/graphdiyne heterostructure (IVR‐FO/GDY). Graphdiyne‐induced iron vacancies can effectively activate the local O sites to transfer electrons towards GDY to boost electrocatalytic nitrogen fixation to ammonia with the high ammonia yield, Faradaic efficiency, as well as the high long‐term stability in neutral electrolytes.

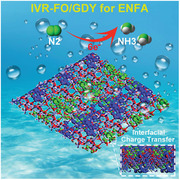

## 1 Introduction

Ammonia, predominately as an important feedstock in agricultural and industrial production, is of great significance to the development of national economy, agriculture and energy as well as people's livelihood. Heavy pollution has become a major burden in modern society, however, Haber–Bosch process is the most common industrial method for industrial ammonia production from nitrogen and hydrogen. However, in order to break the inert N≡N bonds, it needs to be conducted at high pressures (200 atm) and temperatures (400–450 °C), which has caused energy waste and environmental pollution problems. Electrocatalytic nitrogen fixation to ammonia (ENFA) at ambient conditions has been emerging as a most promising alternative to Haber–Bosch process and has occupied a pivotal position in fundamental catalysis research over the years.^[^ ^1^, ^2^, ^3^ ^]^ Efficient ENFA process relies on electrocatalysts with high selectivity, efficiency, and stability, which can efficiently convert the thermostable and chemical inert N_2_ to NH_3_ at ambient conditions.^[^ ^2^, ^4^ ^]^ Construction of vacancy defects on the electrocatalysts has shown to be capable of initiating N≡N activation for ammonia synthesis.^[^ ^5^ ^]^ During past years, various electrocatalysts containing vacancy defects have been reported for the ENFA.^[^ ^5^, ^6^, ^7^, ^8^ ^]^ However, they focus on anion vacancy (for example, oxygen, nitrogen, and carbon vacancies) engineering on electrocatalysts. Until now, it is still rarely reported in controllable fabrication of cation vacancies for efficient ENFA.

Graphdiyne (GDY), an emerging 2D carbon allotrope,^[^ ^9^, ^10^ ^]^ which features unevenly distributed surface charge, abundant porous structure, and high chemical stability, has been proven to be an ideal system for catalysis, energy conversion, and storage.^[^ ^11^, ^12^, ^13^, ^14^, ^15^ ^]^ Unlike conventional carbon‐based electrocatalysts, which are constructed by combining intrinsically inactive carbon materials with metals with finite active sites and low catalytic performances, GDY can effectively regulate morphology, coordination environment and valence states of the metal species, generating new active sites, enhancing electron transfer, and boosting the excellent catalytic activity.^[^ ^14^, ^15^, ^16^ ^]^ Therefore, rational and precise design and fabrication of novel GDY‐based heterogeneous catalysts can be realized by utilizing the advantageous properties of GDY and, consequently, breakthroughs in defect engineering research for high‐efficiency ENFA would be possible.

**Scheme 1:**
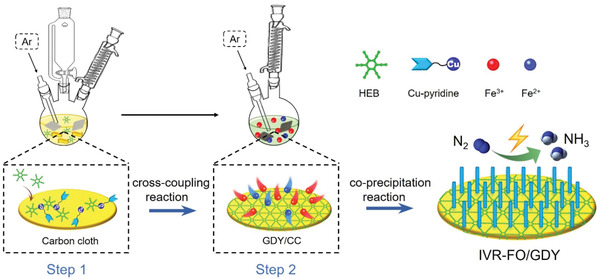
Schematic representation of the synthesis of the IVR‐FO/GDY iron vacancy‐rich catalyst.

Here, we report a facile and effective method to fabricate iron vacancy (*V*_Fe_)‐rich ferroferric oxide on GDY (IVR‐FO/GDY, mass loading: 0.246 mg cm^−2^) for high‐efficiency and ‐selectivity ENFA (**Scheme** **1**). The crystalline structure and valence state of IVR‐FO/GDY were precisely characterized through scanning electron microscopy (SEM), transmission electron microscopy (TEM), X‐ray diffraction (XRD) analysis, Raman spectroscopy, in‐situ and ex‐situ X‐ray absorption spectroscopy (XAS), and X‐ray photoelectron spectroscopy (XPS). It was experimentally determined that by incorporating GDY with Fe_3_O_4_, Fe vacancies could be generated in the crystalline structure. Moreover, the impressive activity of this catalyst was stimulated. The high Y_NH3_ (127.92 ± 9.11 µg h^−1^ mg_cat._^−1^) and FE (59.48 ± 2.57%) of IVR‐FO/GDY far exceeded that of all previously reported Fe‐based catalysts^[^ ^17^, ^18^, ^19^, ^20^ ^]^ as well as most other types of catalysts for the ENFA.^[^ ^8^, ^17^, ^21^, ^22^, ^23^, ^24^, ^25^, ^26^, ^27^, ^28^, ^29^, ^30^ ^]^ Therefore, the GDY‐incorporated catalyst was considered to be promising for practical applications. Furthermore, DFT calculations revealed the electronic structures in IVR‐FO/GDY, which determined its superior electrocatalytic performance for the ENFA. Distinct defect levels in the structure guarantee efficient electron transfer between Fe_3_O_4_ and GDY to facilitate the initial adsorption of N_2_ and the continuous hydrogenation to NH_3_.

## 2 Results and Discussion

### 2.1 Morphological and Structural Characterizations

**Figure 1:**
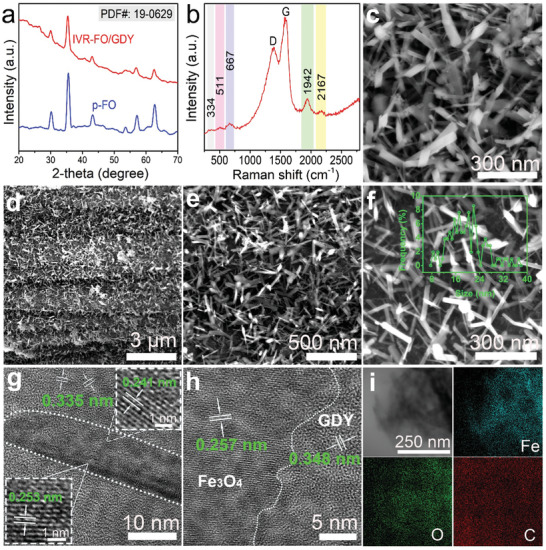
Morphological characterizations. a) Powder XRD patterns of IVR‐FO/GDY and p‐FO. b) Raman spectroscopy of IVR‐FO/GDY. SEM images of c) p‐FO and d–f) IVR‐FO/GDY at different magnifications. g,h) HRTEM images of IVR‐FO/GDY. i) Elemental mapping images of IVR‐FO/GDY.

The crystalline structure of this catalyst was first characterized by XRD, Raman spectroscopy, SEM, and TEM. The powder XRD patterns (**Figure** **1**a) reveals the magnetite spinel phase (PDF: 19–0629) of IVR‐FO/GDY, which is similar to that of pristine Fe_3_O_4_ (p‐FO). The Raman spectra of IVR‐FO/GDY (Figure 1b) and p‐FO (Figure S1, Supporting Information) both exhibit three characteristic peaks corresponding to magnetite.^[^ ^38^ ^]^ Specifically, IVR‐FO/GDY shows two distinct peaks located at 1942 and 2167 cm^−1^, which can be attributed to the vibrations of conjugated diyne links in GDY.^[^ ^9^ ^]^ SEM, TEM, and high‐resolution TEM (HRTEM) were conducted to explore the morphologies and structures of the samples. Figure 1c shows the morphology of the pure Fe_3_O_4_, which exhibits a nanorod structure with irregular width. In contrast, thinner uniform Fe_3_O_4_ nanoneedles (average width: 19.48 nm) form when GDY is introduced as the growing substrate (Figure 1d–f). The lattice fringes of the GDY‐incorporated Fe_3_O_4_ (Figure 1g,h) exhibit visible lattice distortion, which might be due to the defects in catalyst and the strain effect caused by the interaction between Fe_3_O_4_ and GDY. The 0.241, 0.253, and 0.257 nm d‐spacings observed in Figure 1g,h can be attributed to the (222) and (311) planes of spinel magnetite. The interplanar distances of 0.335 and 0.348 nm in the outer space in Figure 1g,h can be attributed to the presence of the GDY substrate. Energy‐dispersive X‐ray spectroscopy (EDX) elemental mapping results (Figure 1i) of IVR‐FO/GDY suggest the uniform distribution of Fe, O, and C in the catalyst.

**Figure 2:**
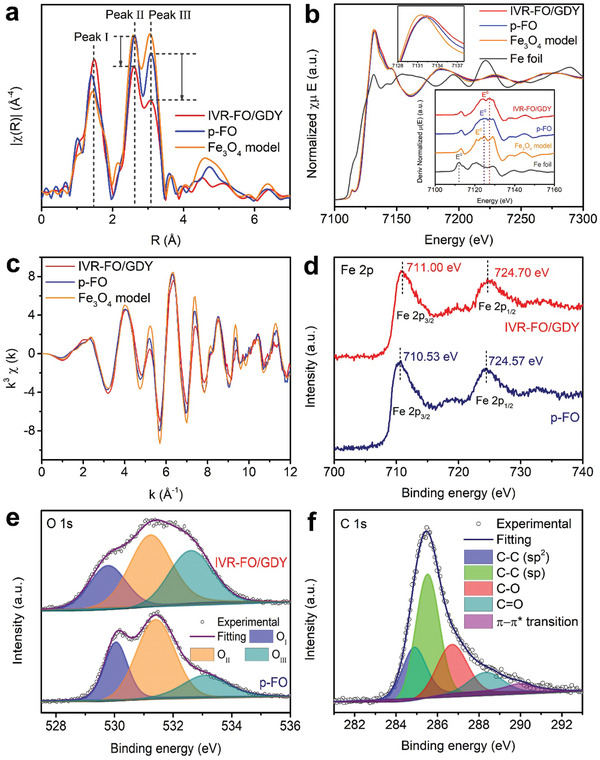
Structural analysis. a) EXAFS spectra of the samples at the Fe K‐edge. b) The normalized Fe K‐edge XANES spectra of different samples (inset at the bottom: the first‐derivative curves of Fe K‐edge XANES spectra of samples). c) Fe K‐edge extended XANES oscillation functions k^3^ *χ*(k). d) Fe 2p XPS spectra of IVR‐FO/GDY and p‐FO. e) O 1s XPS spectra of IVR‐FO /GDY and p‐FO. f) C 1s XPS spectrum of IVR‐FO/GDY.

Extended X‐ray absorption fine structure (EXAFS) analysis was performed to investigate the finer crystalline structure of IVR‐FO/GDY. The EXAFS spectra (**Figure** **2**a) contain three peaks located at ≈1.47, ≈2.63, and ≈3.08 Å, which can be assigned to oxygen‐bonded Fe atoms (Fe—O), octahedral Fe atoms (B‐site), and tetrahedral Fe atoms (A‐site), respectively.^[^ ^39^ ^]^ The visibly weaker amplitudes of the peaks attributed to the Fe—Fe shell suggest an increase of *V*_Fe_ for both the octahedral and tetrahedral sites in IVR‐FO/GDY, with a substantial reduction of 18.8% in the coordination number of the Fe—Fe shell in IVR‐FO/GDY compared with p‐FO (Table S1, Supporting Information). Furthermore, the richer *V*_Fe_ for IVR‐FO/GDY was indicated by its higher Debye–Waller factor, implying an increased degree of disorder and the existence of structural distortion and dangling bonds around the Fe atoms.^[^ ^31^, ^32^ ^]^

Moreover, the chemical state of IVR‐FO/GDY was carefully investigated through the Fe K‐edge X‐ray absorption near edge spectroscopy (XANES) and the corresponding oscillation curves as well as XPS. The different intensities of the XANES spectra for different samples (Figure 2b) primarily arise from the changes in the electronic structures. Furthermore, the greatest absorption edge of IVR‐FO/GDY, obtained from the maximum of its first‐derivative XANES curve, implies a reduction in the electron density of the Fe atoms and a larger proportion of higher‐valence‐state Fe atoms, compared with those of p‐FO and the Fe_3_O_4_ model. The corresponding oscillation curve of IVR‐FO/GDY exhibits a decreased oscillation amplitude relative to that of p‐FO, further verifying their different Fe coordination environments.^[^ ^31^, ^32^, ^33^ ^]^ The XPS survey spectra of the samples are shown in Figure S2a, Supporting Information. The Fe 2p XPS spectra (Figure 2d) of IVR‐FO/GDY and p‐FO demonstrate that more high‐valence Fe atoms exist in IVR‐FO/GDY and this is indicated by the higher binding energies of Fe 2p_3/2_ (711.00 eV) and Fe2p_1/2_ (724.70 eV). The high‐resolution O 1s XPS spectra displayed in Figure 2e show three sub‐peaks attributed to O^2−^ ions bonded to the metals (O_I_), O atoms with dangling bonds (O_II_), and surface‐adsorbed O atoms (O_III_).^[^ ^33^, ^34^, ^35^ ^]^ The higher content of O atoms with dangling bonds in IVR‐FO/GDY also demonstrates its richer *V*_Fe_, which agrees with the best EXAFS fitting results shown in Table S1, Supporting Information. The five sub‐peaks in the C 1s XPS spectrum of IVR‐FO/GDY were assigned to C—C (sp^[^ ^2^ ^]^), C—C (sp), C—O, C═O, and *π*–*π* ^*^ transition^[^ ^9^ ^]^ (Figure 2f). The existence of a *π*–*π* ^*^ transition peak in the spectrum of IVR‐FO/GDY in comparison with that of pristine GDY (Figure S2b, Supporting Information), implies an interaction between the Fe_3_O_4_ and GDY.

### 2.2 Electrocatalytic Nitrogen Fixation to Ammonia Performance

**Figure 3:**
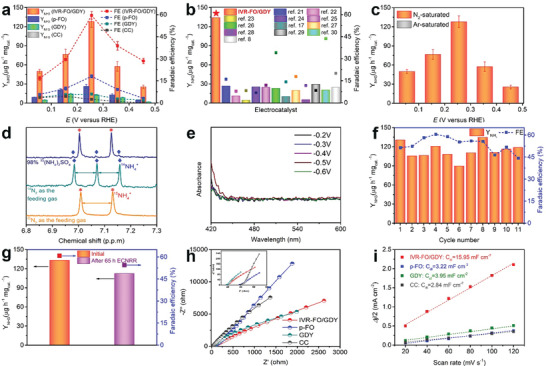
ENFA performances. a) Y_NH3_ and FEs of the samples at different potentials in N_2_‐saturated 0.1 m Na_2_SO_4_ (bars present standard deviation). b) Comparison of Y_NH3_ and FEs of IVR‐FO/GDY with reported ones in neutral condition. c) Y_NH3_ and FEs of IVR‐FO/GDY obtained at different potentials in Ar‐ and N_2_‐saturated 0.1 m Na_2_SO_4_, respectively (bars present standard deviation). d) ^1^H NMR analysis of the electrolytes after ENFA fed by ^15^N_2_ and ^14^N_2_ in 0.1 m Na_2_SO_4_. e) Detection of N_2_H_4_ for IVR‐FO/GDY at applied potentials in N_2_‐saturated 0.1 m Na_2_SO_4_. f) Y_NH3_ and FEs of IVR‐FO/GDY obtained after cycling test at 0.255 V versus RHE in 0.1 m Na_2_SO_4_. g) Y_NH3_ and FEs of IVR‐FO/GDY at 0.255 V versus RHE in 0.1 m Na_2_SO_4_ before and after long‐time stability test. h) Nyquist plots of samples in 0.1 m Na_2_SO_4_ (Inset: corresponding fitted Nyquist plots.). i) Estimated *C*_dl_ values of the samples.

Furthermore, the ENFA performance of the as‐prepared samples was investigated under neutral conditions (Figures S3 and S4, Supporting Information). We equipped a gas purification set‐up to for the electrochemical nitrogen reduction reaction, which has been schematically displayed in Figure S5, Supporting Information. Before each measurement, the feeding gas (including ^14^N_2_, ^15^N_2_, and Ar) was carefully purified through the Cu‐trap to remove the possible impurities such as NO*_x_* and other labile nitrogen compounds. Details regarding the measurements can be found in the Supporting Information. **Figure** **3**a shows the Y_NH3_ and FEs of IVR‐FO/GDY, p‐FO, GDY, and CC at various applied potentials. The corresponding UV–vis curves and chronoamperometry curves are shown in Figure S6, Supporting Information. IVR‐FO/GDY performs remarkably in the ammonia synthesis at a low applied potential of 0.255 V (versus RHE), resulting in an ammonia yield and FE of 127.92 ± 9.11 µg h^−1^ mg_cat_.^−1^ and 59.48 ± 2.57%, respectively; these are much higher than that achieved by the other samples tested at all potentials (Figure 3a and Figure S6c and Table S2, Supporting Information), outperforming most reported ENFA electrocatalysts (Figure 3b and Table S3, Supporting Information).^[^ ^8^, ^17^, ^21^, ^22^, ^23^, ^24^, ^25^, ^26^, ^27^, ^28^, ^29^, ^30^ ^]^ To verify that the detected ammonia originated from nitrogen reduction rather than any contamination, the electrochemical measurements were performed under Ar atmosphere and ^15^N‐isotopic labelling experiments were conducted. Ammonia was not formed at any of the applied potentials under an Ar atmosphere (Figure 3c and Figure S7, Supporting Information). Furthermore, the ^1^H NMR spectrum of the electrolyte after the ENFA using ^15^N_2_ as the feeding gas shows only two split doublets in the range of 6.85–7.15 ppm, which correspond to the characteristic peaks of ^15^NH_4_^+^.^[^ ^12^, ^22^, ^36^, ^37^ ^]^ In contrast, the emerging of the typical triplets assigned to ^14^NH_4_^+^ were observed when the reaction was conducted in the presence of ^14^N_2_ gas (Figure 3d). Besides, quantitative comparison of concentrations of ^14^NH_4_^+^ and ^15^NH_4_^+^ using NMR spectroscopy was conducted and the calculated yields were 127.92 and 121.48 µg h^−1^ mg_cat_.^−1^, suggesting the consistent ammonia yields when ^14^N_2_ and ^15^N_2_ were applied as the feeding gas, respectively (Figure S8, Supporting Information). These results confirm that the detected NH_3_ was generated through electrochemical N_2_ reduction but not due to any possible contamination. Figure 3e shows that no by‐product (N_2_H_4_) was detected at any of the applied potentials, indicating that the as‐prepared IVR‐FO/GDY exhibits 100% selectivity towards the reduction of nitrogen to ammonia. The Y_NH3_ and FE values of IVR‐FO/GDY remained almost constant after a 65‐hour stability test (Figure 3g and Figure S9, Supporting Information). Moreover, IVR‐FO/GDY could well retain its morphology during an 11‐cycle durability test (Figure 3f and Figure S10, Supporting Information), demonstrating the high stability of IVR‐FO/GDY toward ENFA. Electrochemical impedance spectroscopy (EIS) and electrochemical active surface area (ECSA) analysis (Figure S11, Supporting Information) were conducted to determine the origin of the catalytic activity. The fitted electrochemical impedance spectroscopy (EIS) parameters (Table S4, Supporting Information) suggest that IVR‐FO/GDY exhibits the lowest electrochemical impedance among all tested samples, indicating its optimal charge transfer kinetics. Moreover, as shown in Figure 3i, IVR‐FO/GDY shows the highest capacitance of 15.95 mF cm^−2^, which implies that IVR‐FO/GDY has the largest electrochemical active surface area (ECSA) during the electrochemical reaction and, thus, the most available active sites during the ENFA process.

**Figure 4:**
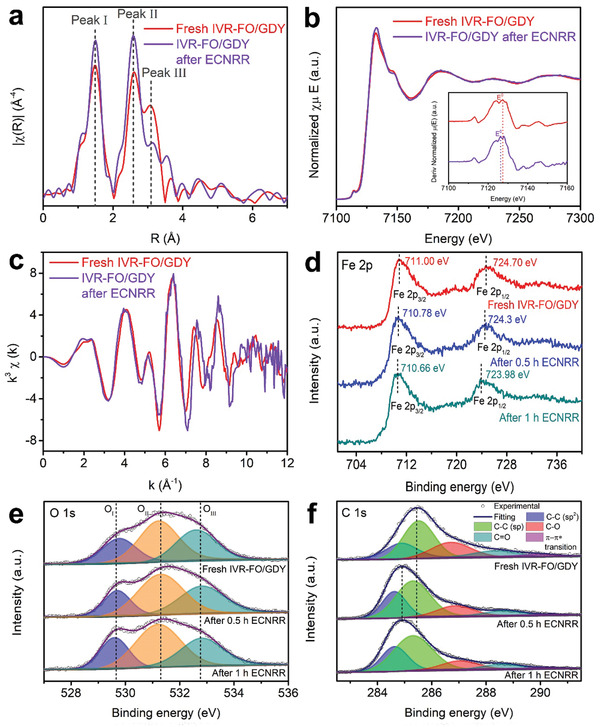
a) In‐situ Fe K‐edge EXAFS spectra of the freshly prepared IVR‐FO/GDY and that obtained after electrocatalysis. b) In‐situ Fe K‐edge XANES spectra of IVR‐FO/GDY before and after electrocatalysis (inset: the first‐derivative curves of Fe K‐edge XANES spectra of samples). c) Fe K‐edge extended in‐situ XANES oscillation functions k^3^ *χ*(k). d) Fe 2p, e) O 1s, and f) C 1s in‐situ XPS spectra of IVR‐FO/GDY along with electrocatalysis time.

Furthermore, in‐situ XPS and XAS measurements were conducted to clarify the ENFA reaction mechanism of IVR‐FO/GDY and investigate the structural and valence state changes during the electrocatalytic process. The EXAFS spectra and fitting results (**Figure** **4**a and Table S1, Supporting Information) as well as the Fe K‐edge extended in‐situ XANES oscillation functions k^3^ *χ*(k) (Figure 4c) show that the coordination number of the Fe atoms in the Fe—Fe shell slightly increases after electrocatalysis, indicating a slight reduction in *V*_Fe_ of IVR‐FO/GDY. This corresponds to a shift to the left for the absorption edge of IVR‐FO/GDY, which was revealed by its in‐situ XANES spectra and first‐derivative curves (Figure 4b). This finding agrees with the XPS results (Figure 4d and Figure S10, Supporting Information), wherein slight negative shifts are observed in the Fe 2p XPS spectra (Figure 4d), suggesting that more low‐valence‐state Fe atoms were generated during the ENFA. In addition, as shown in Figure 4e, the proportion of low‐coordination O atoms decreased as the ENFA proceeded, revealing a slight reduction in the low‐coordination‐number Fe atoms in the nearest Fe—O shell of IVR‐FO/GDY. Moreover, as the reaction time increased, the binding energy of C element (Figure 4f) shifted to lower values, indicating the electron‐rich state of GDY and the strong electron transfer capability of the electrocatalyst during the ENFA.

**Figure 5:**
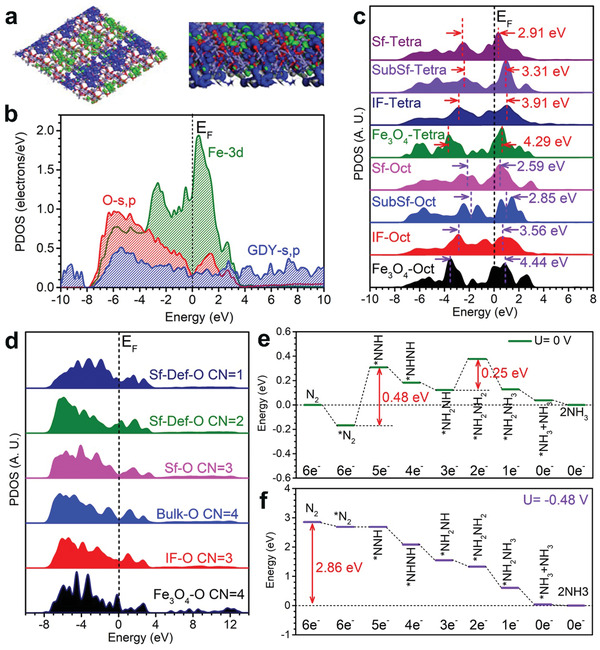
a) 3D real spatial orbital contour plots for bonding and anti‐bonding near the *E*_F_ for IVR‐FO/GDY. b) The PDOSs of IVR‐FO/GDY. c) The site‐to‐site PDOSs of IVR‐FO/GDY. d) The site‐to‐site PDOSs of O‐2p orbitals in IVR‐FO/GDY. e) Energetic pathway of electrocatalysis NRR on IVR‐FO/GDY under *U* = 0 V. f) Energetic pathway of electrocatalysis NRR on IVR‐FO/GDY under *U* = −0.48 V.

Further, we theoretically investigated the IVR‐FO/GDY as an efficient electrocatalyst for the above‐mentioned remarkable NRR performance. The model of IVR‐FO/GDY has been established based on the experimental characterizations, in which more Fe vacancies have been introduced in IVR‐FO/GDY. With the defect generation, electronic distributions have also been varied. Owing to the Fe vacancies, the neighboring O sites became more electron‐rich. From the side view, the strong interfacial charge transfer between Fe_3_O_4_ and GDY was noted, where GDY demonstrated the electron‐rich feature. Notably, the GDY preserved the electron‐rich feature as the electron depletion center for the efficient electron transfer towards adsorbates in IVR‐FO/GDY (**Figure** **5**a). Its projected partial density of states (PDOS) displayed the highly efficient electron transfer capability near the Fermi level (*E*_F_) due to the coupling between GDY and Fe_3_O_4_. We noticed the limited change of the s,p orbitals of GDY, supporting the maintenance of high electroactivity. Meanwhile, the defects still slightly perturbed the electronic structures of local Fe and O sites, which both preserved the high electron transfer capability in IVR‐FO/GDY (Figure 5b). Then, we further looked into the site‐to‐site electronic structures of IVR‐FO/GDY. Both the octahedral and tetrahedral Fe sites have displayed an evident t_2g_–e_g_ splitting in Fe‐3d orbitals. Compared to the pristine Fe_3_O_4_, it was noted that the t_2g_–e_g_ splitting has been alleviated to 2.59 and 2.91 eV for octahedral and tetrahedral Fe sites, respectively, indicating promotion of the electron transfer efficiency (Figure 5c). Meanwhile, with the formation of *V*_Fe_, more O sites became lower coordinated in the structure. From the interface to the surface O sites near the defects, the electron density near *E*_F_ has been increased to facilitate the electron transfer (Figure 5d).

Then, the energy pathways of NRR under electrocatalysis were illustrated. Under the equilibrium potential (*U* = 0 V), we noticed the largest energy barrier of 0.48 eV for the first hydrogenation step from N_2_* to *NNH, which was the rate determining step (RDS), supporting an applied potential similar to the experimental result. Another small energy barrier of 0.25 eV was identified for the transformation from *NH_2_NH to *NH_2_NH_2_. The subsequent hydrogenation and the final desorption of NH_3_ were energetically favorable, confirming the efficient production of NH_3_ (Figure 5e). With the applied potential of −0.48 eV, we noticed a continuous downhill trend for the NRR process without the applied potential. The NRR process released overall energy of 2.86 eV. It was noted that the initial adsorption of N_2_ and the final desorption of NH_3_ were both energetically favorable, which guaranteed the efficient process of NRR (Figure 5f).

## 3 Conclusion

In summary, we proposed a cation‐vacancy‐enabled activation strategy for the electrochemical synthesis of ammonia, and successfully obtained a structure‐optimized Fe‐vacancy‐rich ferroferric oxide/graphdiyne heterostructure. Owing to the synergy between GDY and Fe_3_O_4_, abundant Fe vacancies were introduced into the crystalline structure and the Fe valence state was accurately regulated in IVR‐FO/GDY, therefore improving the efficiency, selectivity, and stability of IVR‐FO/GDY during the ENFA. DFT calculations indicated that the vacancies played a significant role in accelerating nitrogen reduction during the electrocatalytic process. The interfacial coupling and vacancies in the Fe_3_O_4_ promoted significant electron transfer in the composite catalyst, thus benefiting to the efficient adsorption/desorption for electrocatalysis, resulting in impressive ENFA performance. The strategy presented in this work provides a new insight into the design and fabrication of novel catalysts for ammonia synthesis under ambient conditions as well as other energy conversion reactions.

## Conflict of Interest

The authors declare no conflict of interest.

## Supporting information

Supporting Information

## Data Availability

Research data are not shared.
