# Supplementary material for: Graphdiyne‐Induced Iron Vacancy for Efficient Nitrogen Conversion
Source: Adv Sci (Weinh). 2021 Nov 7;9(2):2102721. doi: 10.1002/advs.202102721 (PMC8805558; doi:10.1002/advs.202102721)
Supplement: Supplementary file 1 — Supporting Information [file ADVS-9-2102721-s001.pdf]

ADVANCED SCIENCE
Open Access

Supporting Information

for *Adv. Sci.*, DOI: 10.1002/advs.202102721

Graphdiyne-Induced Iron Vacancy for Efficient Nitrogen Conversion

Yan Fang, Yurui Xue, Lan Hui, Huidi Yu, Chao Zhang, Bolong Huang* and Yuliang Li**

Supporting Information

Graphdiyne-Induced Iron Vacancy for Efficient Nitrogen Conversion

Yan Fang, Yurui Xue, Lan Hui, Huidi Yu, Chao Zhang, Bolong Huang* and Yuliang Li**

Y. Fang, Prof. Y. Xue, L. Hui, H. Yu, C. Zhang, Prof. Y. Li
Institute of Chemistry, Chinese Academy of Sciences, Beijing 100190, P.R. China.
E-mail: yrxue@sdu.edu.cn; ylli@iccas.ac.cn

Prof. Y. Xue
Science Center for Material Creation and Energy Conversion, Institute of Frontier and Interdisciplinary Science, School of Chemistry and Chemical Engineering, Shandong University, Jinan 250100, P.R. China

Prof. B. Huang
Department of Applied Biology and Chemical Technology, the Hong Kong Polytechnic University, Hung Hom, Kowloon, Hong Kong SAR, P.R. China.
E-mail: bhuang@polyu.edu.hk

Y. Fang, L. Hui, H. Yu, C. Zhang,Prof. Y. Li
University of Chinese Academy of Sciences, Beijing 100049, P. R. China

Materials

$FeSO_4 \cdot 7H_2O$ and $FeCl_3 \cdot 6H_2O$ were both purchased from Energy Chemical. Hexabromobenzene and Tetrabutylammonium fluoride (TBAF) were provided by J&K Scientific and Alfa Aesar, respectively. Tetrahydrofuran (THF) and toluene were both refluxed with sodium to remove the residual water before utilization. Water used in this experiment was purified with a Millipore system. All the other reagents were both utilized as received without any purification unless specifically illustrated.

Experimental Section

Preparation of IVR-FO/GDY: The GDY/CC was firstly synthesized through a modified Glaser-Hay coupling reaction as the growing substrate for ferroferric oxide. Briefly, several carbon cloth (CC) pretreated by nitric acid and copper foils were immersed in a solution containing acetone, pyridine and N,N,N',N'-tetramethylethylenediamine (TMEDA) with a volume ratio of 100: 5:1 and the system was heated up to 50 ˚C. 50 mL acetone solution containing 50 mg hexakis[(trimethylsilyl)ethynyl]benzene (HEB) was added to the solution dropwise and the reactor was kept at 50 ˚C for 24 h under Ar protection. Then the as-prepared GDY/CC was taken out from the reactor and washed with N,N-Dimethylformamide (DMF), acetone successivly and then immersed in 0.5 M HCl to remove the residual Cu species. Finally, the samples were washed with deionized water and ethanol for several times and dried in atmosphere at room temperature.

IVR-FO/GDY was then prepared via a mild and facile co-precipitation method. Firstly, three pieces of freshly prepared GDY/CC were immersed in 150 mL aqueous solution containg 3.5 mM $FeSO_4 \cdot 7H_2O$ and 7.1 mM $FeCl_3 \cdot 6H_2O$ for 1 h under Ar protection, allowing the adsorption of iron species. The above solution was then heated up to 80 ˚C and 10 mL 2.8 M KOH aqueous solution was quickly injected into the solution. The reactor was maintained at 80 ˚C for 2 h under mild stirring. Finally, the synthesized IVR-FO/GDY was rinsed with

deionized water and ethanol for several times and dried in vacuum at 25 ˚C and kept under Ar atmosphere.

p-FO was synthesized through the same method but without the utilization of GDY/CC.

Electrocatalytic measurements: We equipped a gas purification set-up to conduct the electrochemical nitrogen reduction experiment according to *Nature* **2019**, *570*, 504 (as can be seen in **Figure S**4). Before each test, the feeding gas (including $^{14}N_2$, $^{15}N_2$ and Ar) was carefully purified to remove any possible impurities such as NO_x and other labile nitrogen compounds in order to mitigate the contributions of contaminates. All the electrochemical measurements were performed on a typical three-electrode workstation (CHI. 660D, Shanghai CH. Instruments, China). The nitrogen reduction reaction was carried out in 0.1 M Na_2SO_4 (pH=7) in a H-type cell separated by Nafion 117 membrane under ambient pressures and temperatures. The Nafion 117 membranes used in this experiment were protonated by boiling in water for 1 h, in H_2O_2 for 1 h and then in water for another 1 h, followed by being boiled in 0.5 M H_2SO_4 for 3 h and in water for 6 h before measurement. All the boiling steps above were performed at 80 °C. 0.1 M Na_2SO_4 aqueous solution was prepared as the electrolyte, freshly prepared samples were directly utilized as the working electrode, the saturated calomel electrode (SCE) was used as the reference electrode, and the graphitic rod was used as the counter electrode. ENFA was firstly performed at a range of potentials in the N_2-saturated electrolyte with high-purity $^{14}N_2$ continuously bubbled to the surface of cathode for 1 h and then the electrolytes were collected for products detection (the electrolyte was bubbled with high purity nitrogen for 2 h before electrochemical tests). The ENFA measurements were repeated for 4 times at every applied potential in repetitive tests. In the cycling stability test, electrolytes were collected at an interval of 1 h in order to detect products and the tested samples and the reactor were rinsed with deionized water for several times in order to remove purities and absorbed ammonia on the surface. And then the cleaned samples were immersed in electrolytes again for the next electrocatalysis cycle. The procedures of argon control

experiments were the same as nitrogen reduction experiments except from the feeding gas. And when $^{15}N_2$ was used as the feeding gas, we firstly saturated the electrolyte with high-purity Ar to remove excess $^{14}N_2$ and any other residual gas, followed by bubbling high-purity $^{15}N_2$ to the sealed electrolyte for 30 min and the electrocatalytic reaction was performed at 0.255 V vs. RHE for 1 h, with $^{15}N_2$ continuously being supplied.

Determination of ammonia: The electrocatalytically synthesized ammonia was detected through a standard spectrophotometry method. The corresponding calibration curve was determined as follows: 4 mL standard NH_4Cl solutions with series of concentrations in 0.1 M Na_2SO_4 were mixed with 50 μL oxidizing solution [NaClO (ρCl = 4~4.9) solution containing 0.75 M NaOH], 500 μL coloring solution (aqueous solution of 0.4 M $C_7H_5O_3Na$ and 0.32 M NaOH) and 50 μL catalyst solution (aqueous solution of 1% $Na_2[Fe(NO)(CN)_5]\cdot 2H_2O$) successively. UV-vis measurements were conducted within a range of 800 nm to 500 nm after standing at ambient conditions for 2 hours. The calibration curve was obtained from the absorbance at 660 nm and calculated as: y = 0.1527x + 0.01046, R^2 = 0.999.

Ammonia yield (Y_{NH3}) of samples was evaluated using the following equation:

$$Y_{NH3} = (C_{NH4Cl} \times 0.318 \times V) / (t \times m) \qquad (1)$$

where C_{NH4Cl} is the measured concentration, V is the volume of electrolyte, t is the electrolysis time and m is the mass loading of catalyst.

Faradaic efficiency (FE) of samples in ECNRR was calculated as follows:

$$FE = (3 \times F \times C_{NH3} \times V) / (17 \times Q) \qquad (2)$$

where F is the Faradaic constant, C_{NH3} is the measured concentration of ammonia, V is the volume of electrolyte and Q is the quantity of applied electricity.

Detection of hydrazine: The N_2H_4 was detected by Watt and Chrisp method. 5.99 g ρ-$C_9H_{11}NO$, 30 mL HCl and 300 mL C_2H_5OH were mixed as the color reagent. 2.5 mL

electrolyte after electrocatalysis was mixed with 2.5 mL above color reagent, and then measured by UV-vis spectrophotometer within the range of 600 nm to 400 nm after standing at ambient conditions for 10 minutes.

Characterizations

X-ray diffraction (XRD) was conducted on the Rigaku D/max-2500 rotation anode X-ray diffractometer using Cu Kα radiation (λ=1.54178 Å). Scanning electron microscopy (SEM） images were collected using an S-4800 field emission scanning electron microscope. Transmission electron microscopy (TEM), high-resolution transmission electron microscopy (HRTEM) and energy dispersive X-ray spectroscopy (EDX) results were all carried out through a JEM-2100F electron microscope. X-ray photoelectron spectroscopy (XPS) measurement was performed on a Thermo Scientific ESCALab 250Xi instrument with monochromatic Al Kα. The X-ray absorption fine structure spectra (Fe K-edge) were collected at 1W1B station in Beijing Synchrotron Radiation Facility (BSRF). The storage rings of BSRF was operated at 2.5 GeV with a maximum current of 250 mA. Using Si (111) double-crystal monochromator, the data collection was carried out in transmission mode using ionization chamber. All spectra were collected in ambient conditions. The nuclear magnetic resonance (NMR) experiment was performed on a Bruker AVANCE spectrometer operating at a ^{1}H frequency of 600 MHz. Before NMR measurement, 500 μL electrolyte to be tested was added to the NMR tube and the electrolyte was adjusted to be weakly acid.

XAFS measurements

The X-ray absorption find structure spectra (Fe K-edge) were collected at 1W1B station in Beijing Synchrotron Radiation Facility (BSRF). The storage rings of BSRF were operated at 2.5 GeV with an average current of 250 mA. Using Si(111) double-crystal monochromator,

the data collection were carried out in transmission/fluorescence mode using ionization chamber. All spectra were collected in ambient conditions.

XAFS analysis and results

The acquired EXAFS data were processed according to the standard procedures using the ATHENA module implemented in the IFEFFIT software packages. The k3-weighted EXAFS spectra were obtained by subtracting the post-edge background from the overall absorption and then normalizing with respect to the edge-jump step. Subsequently, k3-weighted χ(k) data of Fe K-edge were Fourier transformed to real (R) space using a hanning windows (dk=1.0 Å-1) to separate the EXAFS contributions from different coordination shells. To obtain the quantitative structural parameters around central atoms, least-squares curve parameter fitting was performed using the ARTEMIS module of IFEFFIT software packages.

Calculation setup

For the calcualtions of both electrocatalysis and photocatalysis, we have introduced the DFT calculation embedded in CASTEP packages [1]. For the functionals, we have selected the generalized gradient approximation (GGA) and the Perdew-Burke-Ernzerhof (PBE) [2-4]. Meanwhile, the cutoff energy of the plane-wave basis is set to be 380 eV with the ultrasoft pseudopotantial scheme [5-6]. The IVR-FO/GDY composite catalysts have been built based on the combination of Fe_3O_4 (311) surface with single layer of GDY. As the experiments characterized, the IVR-FO/GDY contains more Fe vacancies. We have also applied 20 Å vacuum space along z-direction to avoid interactions between periodic lattice. For all the geometry optimization, the Hellmann-Feynman forces should be converged to smaller than 0.001 eV/Å. Meanwhile, the total energy and inter-ionic displacement should not exceed 5×10^{-5} eV/atom and 0.005 Å, respectively. Considering the computational loading and

effficiency, the coarse k-point has been applied for the energy minimization based on the Broyden-Fletcher-Goldfarb-Shannon (BFGS) algorithm [7].

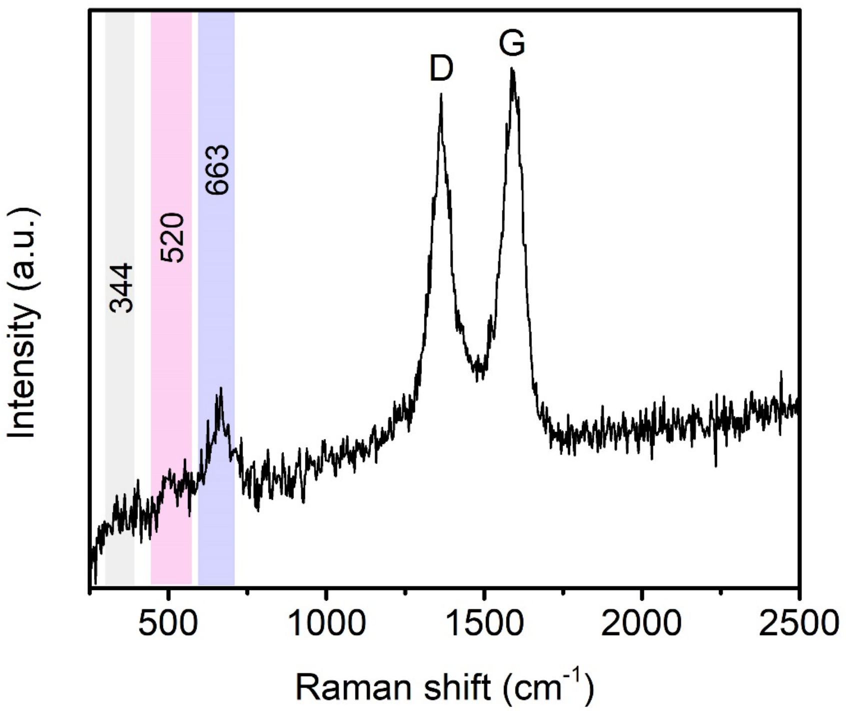

Figure S1. Raman spectroscopy of p-FO loaded on carbon cloth.

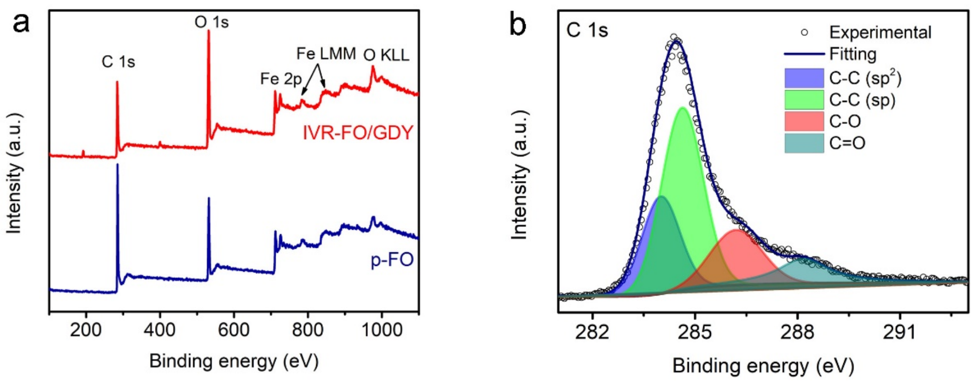

Figure S2. XPS measurements. a) XPS survey spectra of IVR-FO/GDY and p-FO. b) C 1s XPS spectrum of pristine GDY.

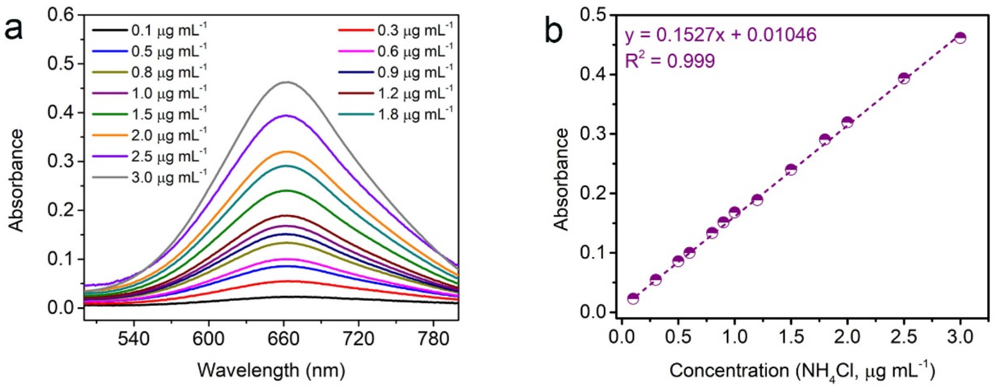

Figure S3. Determination of ammonia. a) UV-vis absorption spectra of indophenol assays with standard NH_4Cl solutions in 0.1 M Na_2SO_4 after 2 h incubation at room temperature. b) The calibration curve used for calculation of NH_3 concentrations in 0.1 M Na_2SO_4.

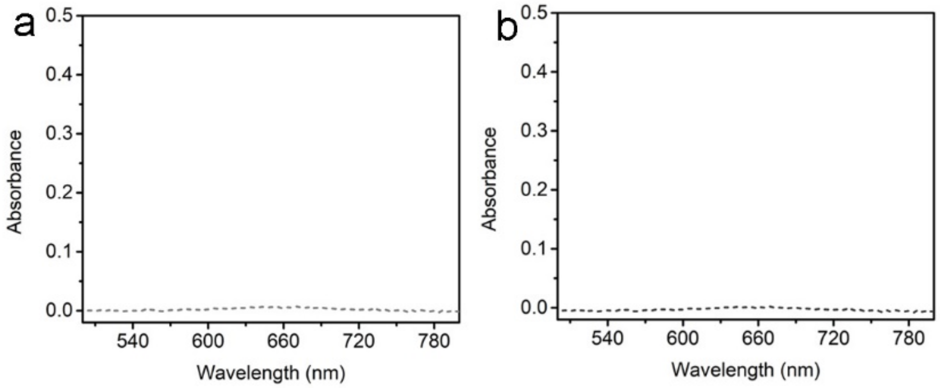

Figure S4. a) UV-vis absorption spectrum of freshly prepared 0.1 M N_2-saturated Na_2SO_4. b) UV-vis absorption spectrum of the electrolyte after testing in an open circuit for IVR-FO/GDY.

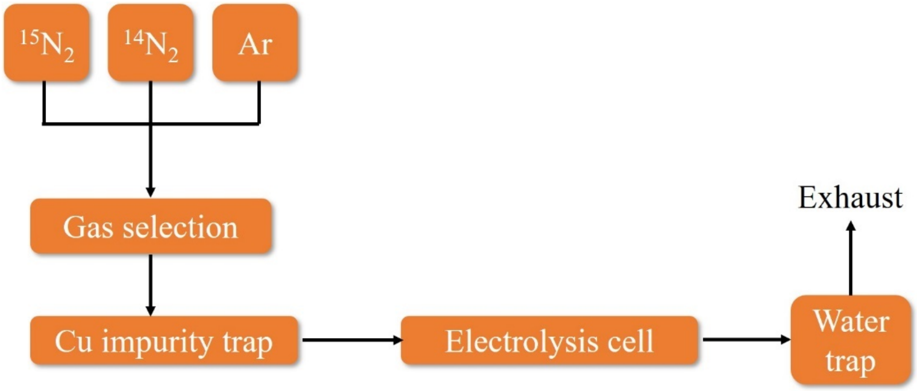

Figure S5. Schematic illustration of the gas purification set-up.

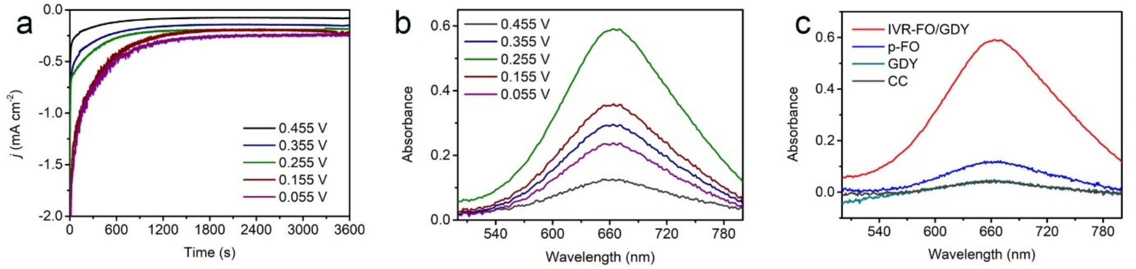

Figure S6. a) Chronoamperometry curves of IVR-FO/GDY tested at different potentials in 0.1 M Na_2SO_4. b) UV-vis absorption spectra of the 0.1 M Na_2SO_4 after ENFA at different potentials for 1 h under ambient conditions. c) UV-vis absorption spectra of the 0.1 M Na_2SO_4 electrolytes after ENFA at 0.255 V versus RHE for all samples.

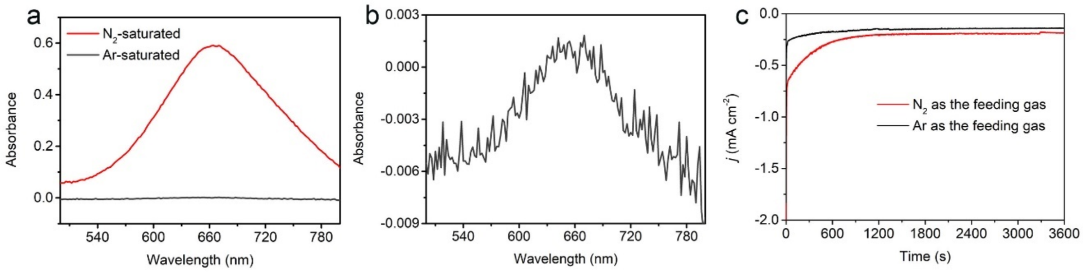

Figure S7. a) UV-vis absorption spectra of the N_2- and Ar-saturated 0.1 M Na_2SO_4 electrolytes after ENFA at 0.255 V versus RHE. b) UV-vis absorption spectra of the Ar-saturated 0.1 M Na_2SO_4 electrolyte after ENFA at 0.255 V versus RHE. c) Chronoamperometry curves of IVR-FO/GDY tested at 0.255 V versus RHE under N_2 and Ar environment respectively in 0.1 M Na_2SO_4.

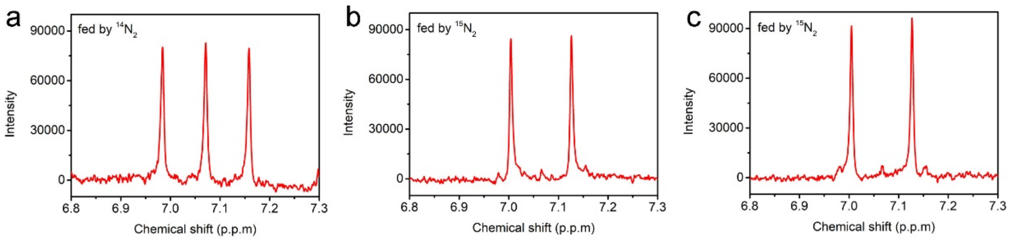

Figure S8. a) NMR data of the electrolyte after ENFA measurement when fed by 14N2. b, c) NMR data of the electrolytes after two repetitive ENFA measurements when fed by 15N2.

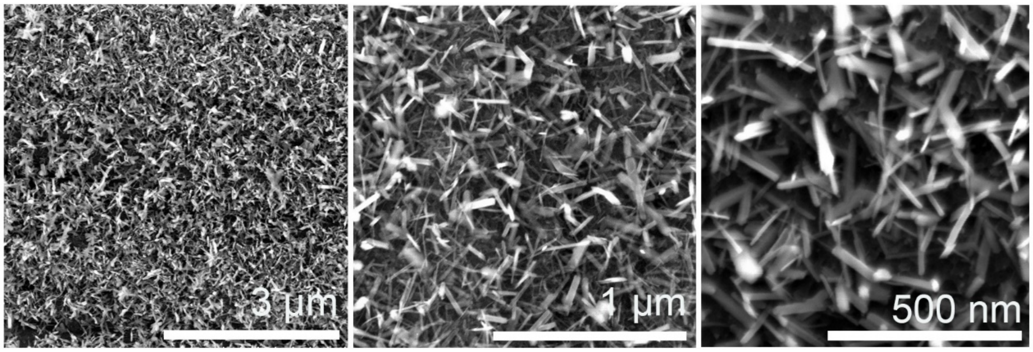

Figure S9. Morphological characterizations of IVR-FO/GDY after cycling tests in 0.1 M Na_2SO_4.

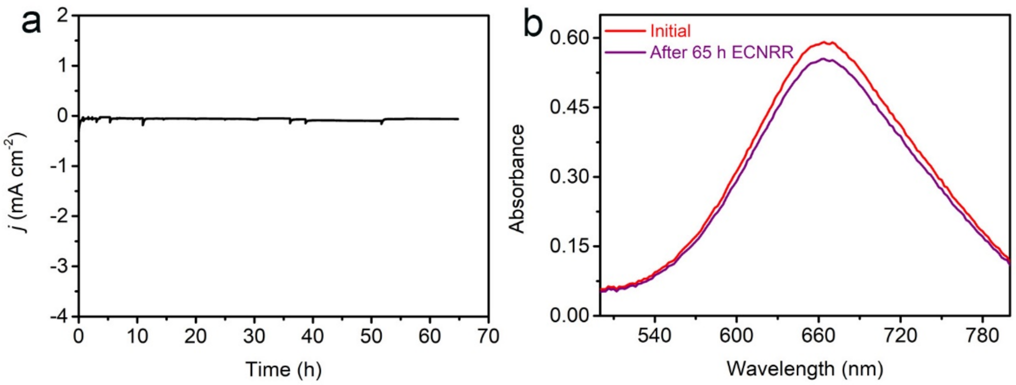

Figure S10. a) Chronoamperometry curve of long-time stability test at 0.255 V versus RHE in 0.1 M Na_2SO_4 for IVR-FO/GDY. b) UV-vis absorption spectra of the 0.1 M Na_2SO_4 electrolytes after ENFA at 0.255 V versus RHE for IVR-FO/GDY.

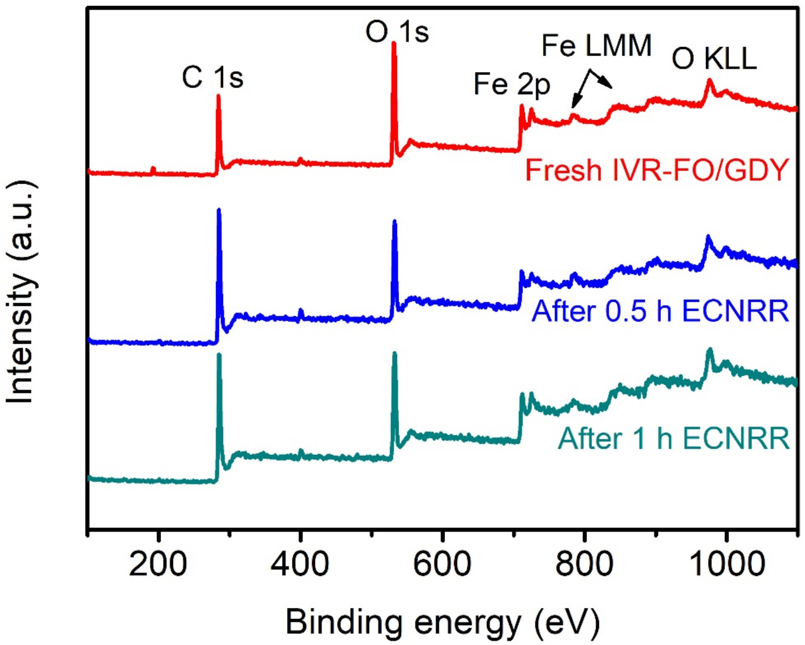

Figure S11. In-situ XPS survey spectra of IVR-FO/GDY along with electrocatalysis time.

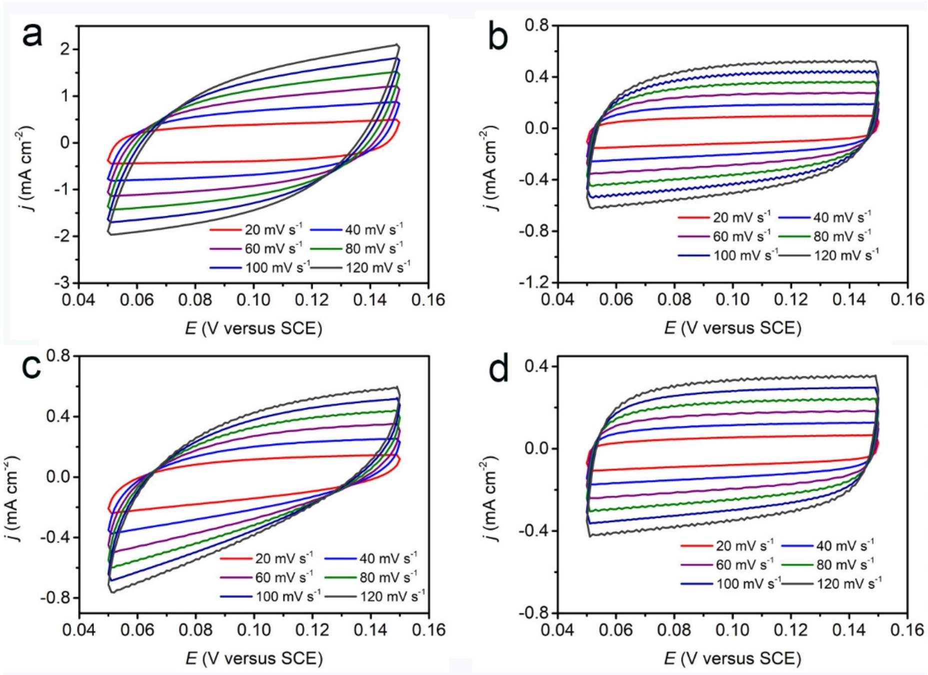

Figure S12. CV curves of a) IVR-FO/GDY, b) p-FO, c) GDY d) CC recorded at the scan rate from 20 to 120 mV s^{-1}.

Table S1. EXAFS fitting parameters at the Fe K-edge for various samples.

Sample	Shell	*N* [a]	*R* (Å) [b]	σ^2 (Å^2·10^3) [c]	*ΔE0 (eV)* [d]	*R factor (%)*
IVR-FO/GDY	Fe-O	4.5	1.96	7.8	0.3	0.2
	Fe-O	1.4	2.12	4.7		
	Fe-O-Fe	3.5	2.98	11.1	1.1	
	Fe-O-Fe	4.7	3.47	8.4		
p-FO	Fe-O	2.2	1.91	4.6	-1.2	0.1
	Fe-O	3.2	2.05	6.5		
	Fe-O-Fe	4.9	2.99	11.4	-2.5	
	Fe-O-Fe	5.2	3.48	7.2		
IVR-FO/GDY after ENFA	Fe-O	2.7	1.92	2.7	-4.1	0.9
	Fe-O	2.1	2.05	3.9		
	Fe-O-Fe	4.4	2.99	9.3	-1.1	
	Fe-O-Fe	4.0	3.33	7.0		

[a] *N*: coordination numbers; [b] *R*: bond distance; [c] σ^2: Debye-Waller factors; [d] ΔE_0: the inner potential correction. *R* factor: goodness of fit. *S*02 were set as 0.84/0.89 for Fe-O/Fe-O-Fe, which was obtained from the experimental EXAFS fit of reference Fe_2O_3 by fixing CN as the known crystallographic value and was fixed to all the samples.

Error range for N and σ^2: 20%. Accuracy range for R: ±0.03 Å.

.

Table S2. Y_{NH3} and FEs of samples for repetitive ENFA tests at respective optimal potential in 0.1 M Na_2SO_4.

**IVR-FO/GDY**	**1**	**2**	**3**	**4**
Y_{NH3} (μg h^{-1} $mg_{cat.}^{-1}$)	114.49	133.08	130.09	134.02
FE (%)	55.63	60.73	60.88	60.69
**p-FO**	**1**	**2**	**3**	**4**
Y_{NH3} (μg h^{-1} $mg_{cat.}^{-1}$)	29.91	27.45	24.73	23.16
FE (%)	18.85	16.99	17.24	19.01
**GDY**	**1**	**2**	**3**	**4**
Y_{NH3} (μg h^{-1} $mg_{cat.}^{-1}$)	14.29	14.22	15.30	13.92
FE (%)	6.30	6.02	5.83	5.70
**CC**	**1**	**2**	**3**	**4**
Y_{NH3} (μg h^{-1} $mg_{cat.}^{-1}$)	0.29	0.3	0.33	0.25
FE (%)	4.56	4.98	3.68	4.43

Table S3. Comparison of Y_{NH3} and FEs of IVR-FO/GDY with previously reported electrocatalysts applied in neutral conditions.

Electrocatalyst	Yield (μg h^{-1} $mg_{cat.}^{-1}$)	FE (%)	Reference
**IVR-FO/GDY**	**134.02**	**60.88**	**This work**
B_4C	14.7	9.24	[8]
Mo_2C/C	11.3	7.8	[9]
Pd/C	4.5	8.2	[10]
Fe/Fe_3O_4	0.19 μg h^{-1} cm^{-2}	8.29	[11]
MnO/TM	1.11×10^{-10} mol h^{-1} cm^{-2}	8.02	[12]
Fe_2O_3/CNT	2.2×10^{-3} g m^{-2} h^{-1}	4.9	[13]
MoS_2/CC	8.08×10^{-11} mol h^{-1} cm^{-2}	1.17	[14]
DR MoS_2	29.2	8.34	[15]
MHCMs	25.3	6.78	[16]
JUC-1000/CC	24.7	11.9	[17]
$PC/Sb/SbPO_4$	23	34	[18]
FeN_4	10.25	14.17	[19]
$FeS@MoS_2/CFC$	8.45 μg h^{-1} cm^{-2}	2.96	[20]
$C\text{-}Ti_xO_y/C$	19.8	17.8	[21]
Plasma R-O-Bi	5.453	11.68	[22]
$N@MoS_2$	69.82	9.14	[23]
np-$PdH_{0.43}$	20.4	43.6	[24]
$W_{18}O_{49}$-16Fe@CFP	24.7	20	[25]

Table S4. Impedance parameters derived from the fitting to the equivalent circuit for the impedance spectra recorded in 0.1 M Na_2SO_4.

Electrocatalyst	Fresh IVR-FO/GDY	p-FO	GDY	CC
R_s (ohm)	17.55	37.37	17.79	38.43
CPE1, Yo (S-sec^n)	8.05×10^{-3}	4.47×10^{-3}	1.92×10^{-2}	5.68×10^{-3}
n	0.57	0.51	0.48	0.47
R_{ct} (ohm)	2.81	7.15	3.56	13.61
CPE2, Yo (S-sec^n)	1.73×10^{-3}	4.67×10^{-4}	2.18×10^{-3}	2.82×10^{-4}
n	0.85	0.87	0.93	0.97
R' (ohm)	1.89×10^{3}	1.42×10^{3}	1.17×10^{3}	6.06×10^{3}

References

[1] S. J. Clark, S. J. Clark, M. D. Segall, C. J. Pickard, P. J. Hasnip, M. J. Probert, K. Refson, M. C. Payne, *Zeitschrift Fur Kristallographie* **2005**, *220*, 567.

[2] J. P. Perdew, K. Burke, M. Ernzerhof, *Phys Rev Lett 77* **1996**, 3865.

[3] J. P. Perdew, M. Ernzerhof, K. Burke, *The Journal of Chemical Physics* **1996**, *105*, 9982.

[4] D. Vanderbilt, *Physical Review B 41* **1990**, 7892.

[5] P. J. Hasnip, C. J. Pickard, *Comput Phys Commun.* **2006**, *174*, 24.

[6] J. P. Perdew, J. A. Chevary, S. H. Vosko, K. A. Jackson, M. R. Pederson, D. J. Singh, C. Fiolhais, *Physical Review B* **1992**, *46*, 6671.

[7] J. D. Head, M. C. Zerner, *Chem Phys Lett* **1985**, *122*, 264.

[8] W. Qiu, X. Xie, J. Qiu, W. Fang, R. Liang, X. Ren, X. Ji, G. Cui, A. M. Asiri, G. Cui, B. Tang, X. Sun, *Nat. Commun.* **2018**, *9*, 3485.

[9] H. Cheng, L. Ding, G. Chen, L. Zhang, J. Xue, h. Wang, *Adv. Mater.* **2018**, 1803694.

[10] J. Wang, L. Yu, L. Hu, G. Chen, H. Xin, X. Feng, *Nat. Commun.* **2018**, *9,* 1795.

[11] L. Hu, A. Khaniya, J. Wang, G. Chen. W. E. Kaden, X. Feng, *ACS Catal.* **2018**, *8*, 9312.

[12] Z. Wang, F. Gong, L. Zhang, R. Wang, L. Ji, Q. Liu, Y. Luo, H. Guo, Y. Li, P. Gao, X. Shi, B. Li, B. Tang, X. Sun. *Adv, Sci.* **2019**, *6*, 1801182.

[13] S. Chen, S. Perathoner, C. Ampelli, C. Mebrahtu, D. Su, G. Centi, *Angew. Chem. Int. Ed.* **2017**, *56*, 2699.

[14] L. Zhang, X. Ji, X. Ren, Y. Ma, X. Shi, Z. Tian, A. M. Asiri, L. Chen, B. Tang, X. Sun, *Adv, Mater.* **2018**, *30*, 1800191.

[15] X. Li, T. Li, Y. Ma, Q. Wei, W. Qiu, H. Guo, X. Shi, P. Zhang, A. M. Asiri, L. Chen, B. Tang, X. Sun, *Adv. Energy Mater* **2018**, *8*, 1801357.

[16] Y. Zhang, W. Qiu, Y. Ma, Y. Luo, Z. Tian, G. Cui, F. Xie, L. Chen, T. Li, X. Sun, *ACS Catal.* **2018**, *8*, 8540.

[17] L. Zhao, X. Kuang, C. Chen, X. Sun, Z. Wang, Q. Wei, *Chem. Commun*. **2019**, *55*, 10170.

[18] X. Liu, H. Jang, P. Li, J. Wang, Q. Qin, M. Kim, G. Li, J. Cho, *Angew. Chem. Int. Ed*. **2019**, *58*, 13329.

[19] C. He, Z. Wu, L. Zhao, M. Ming, Y. Zhang, Y. Yi, J. Hu, *ACS Catal*. **2019**, *9*, 7311.

[20] Y. Guo, Z. Yao, B. J. J. Timmer, X. Sheng, L. Fan, Y. Li, F. Zhang, L Sun, *Nano Energy*. **2019**, *62*, 282.

[21] Q. Qin, Y. Zhao, M. Schmallegger, T. Heil, J. Schmidt, R. Walczak, G. Gescheidt-Demner, H. Jiao, M. Oschatz, *Angew. Chem. Int. Ed*. **2019**, *58*, 13101.

[22] Y. Wang, M. Shi, D. Bao, F. Meng, Q. Zhang, Y. Zhou, K. Liu, Y. Zhang, J. Wang, Z. Chen, D. Liu, Z. Jiang, M. Luo, L. Gu, Q. Zhang, X. Cao, Y. Yao, M. Shao, Y. Zhang, X. Zhang, J. G. Chen, J. Yan, Q. Jiang, *Angew. Chem. Int. Ed*. **2019**, *58*, 9464.

[23] L. Zeng, S. Chen, J. Zalm, X. Li, A. Chen, *Chem. Commun*. **2019**, *55*, 7386.

[24] W. Xu, G. Fan, J. Chen, J. Li, L. Zhang, S. Zhu, X. Su, F. Cheng, J. Chen, *Angew. Chem. Int. Ed*. **2020**, *59*, 3511.

[25] Y. Tong, H. Guo, D. Liu, X. Yan, P. Sun, J. Liang, S. Zhou, J. Liu, G. Qing. S. Dou, *Angew. Chem. Int. Ed*. **2020**, *59*, 7356.
